# Parameters optimization method for the time-delayed reservoir computing with a nonlinear duffing mechanical oscillator

**DOI:** 10.1038/s41598-020-80339-5

**Published:** 2021-01-13

**Authors:** T. Y. Zheng, W. H. Yang, J. Sun, X. Y. Xiong, Z. T. Li, X. D. Zou

**Affiliations:** 1 The State Key Laboratory of Transducer Technology, Aerospace Information Research Institute, Chinese Academy of Sciences, Beijing, 100000 China; 2 School of Electronic, Electrical and Communication Engineering, University of Chinese Academy of Sciences, Beijing, 100000 China

**Keywords:** Computational science, Applied physics

## Abstract

Reservoir computing (RC) is a recently introduced bio-inspired computational framework capable of excellent performances in the temporal data processing, owing to its derivation from the recurrent neural network (RNN). It is well-known for the fast and effective training scheme, as well as the ease of the hardware implementation, but also the problematic sensitivity of its performance to the optimizable architecture parameters. In this article, a particular time-delayed RC with a single clamped–clamped silicon beam resonator that exhibits a classical Duffing nonlinearity is presented and its optimization problem is studied. Specifically, we numerically analyze the nonlinear response of the resonator and find a quasi-linear bifurcation point shift of the driving voltage with the driving frequency sweeping, which is called Bifurcation Point Frequency Modulation (BPFM). Furthermore, we first proposed that this method can be used to find the optimal driving frequency of RC with a Duffing mechanical resonator for a given task, and then put forward a comprehensive optimization process. The high performance of RC presented on four typical tasks proves the feasibility of this optimization method. Finally, we envision the potential application of the method based on the BPFM in our future work to implement the RC with other mechanical oscillators.

## Introduction

Efficient information processing is among the most prominent challenges in modern society. Its significance becomes immediately apparent considering advances made in the wake of emerging applications of sensors, such as the Internet of Things (IOT) and ubiquitous sensing. The massive raw data generated by thousands sensor nodes is consuming more and more transmission bandwidth, storage capacity, and energy, which calls for systems capable of processing information efficiently with low power consumption. The increase in need for information processing capacity, as well as the physical limitations of the Turing or von Neumann machine methods implemented in most computational systems, have motivated the search for novel computational paradigms some of which present an outstanding potential. One approach is Reservoir computing (RC), a bio-inspired computational framework with high energy efficiency while avoiding Von Neumann bottleneck, which has witnessed remarkable progress in recent years^1–4^.

Reservoir computing is originally a recurrent neural network (RNN) framework and is therefore suitable for temporal information processing^5,6^. An RC system usually consists of an input layer, a dynamical Reservoir layer, and an output layer. The Reservoir is usually a network of randomly connected nonlinear nodes and nonlinearly transforms sequential inputs into a high-dimensional space such that the features of the inputs can be efficicently read out by a simple learning algorithm such as linear regression. During the training phase, the Reservoir is fixed and only the output weight is trained, which makes it faster and more efficient than other RNNs. More importantly, the fixed Reservoir without adaptive updating is amenable to hardware implementation using a variety of nonlinear dynamical systems^7,8^. Comparatively, RC based on the time-delayed nonlinear system greatly reduces the implementation difficulty, which has attracted increasing attention in diverse fields of research, such as electronic^9–11^, spintronic^12–14^, optoelectronic^15–17^, and all-optical^18–20^ delay systems as well as mechanical resonator^21,22^. The success of these systems demonstrated that RC based on a nonlinear system with time-delay feedback can achieve excellent performance on time-dependent signal recognition tasks such as speech recognition and time-series prediction. Although these previous works demonstrated the success of hardware RC implementation based on a nonlinear system with time-delay feedback and provided significant gains over conventional computing paradigms in terms of speed or energy efficiency, the performance improvement by optimizing the parameters of the time-delayed systems could be envisioned. Remarkably, the implementation of RC using the non-linear dynamics of a single clamped–clamped silicon beam driven electrostatically at large oscillation amplitudes has been first demonstrated experimentally by Julien Sylvestre’s group^21^, which shed light on the potential of MEMS-based RC and provides a platform for the optimization research of RC with a nonlinear mechanical oscillator.

In this vein, we are inspired to present a time-delayed hardware RC with a single clamped–clamped silicon beam resonator that exhibits a classical Duffing nonlinearity. To efficiently optimize the RC described for given tasks, we numerically analyze the nonlinear response of the resonator and study the relationship between the bifurcation point of the driving voltage and the driving frequency. Then we find a quasi-linear bifurcation point shift with the frequency sweeping, which is called Bifurcation Point Frequency Modulation (BPFM). Moreover, we first proposed that this method can be used to find the optimal driving frequency of RC with a Duffing mechanical resonator for a given task, and further put forward a comprehensive optimization process. To prove that, we investigate the performance of the RC presented with the method of BPFM on four typical tasks such as the signal classification, isolated spoken digits recognition, Parity benchmark, and nonlinear autoregressive moving average (NARMA10) task. The high performance proves the feasibility of this optimization method. Besides, we discuss the influence of some other typical parameters of the RC we presented. Finally, we envision the potential application of the method based on the BPFM in our future work to implement the time-delayed RC with other mechanical oscillators.

The remainder of this paper is organized as follows, first, we present the structure of the time-delayed RC, the sequence of parameters optimization, and the analysis of the BPFM method. And then, we testify the numerical results of our system on four typical tasks. Finally, we discuss the conclusion and the outlook for possible future work.

## Theory and analysis

### The structure of the reservoir computing

**Figure 1:**
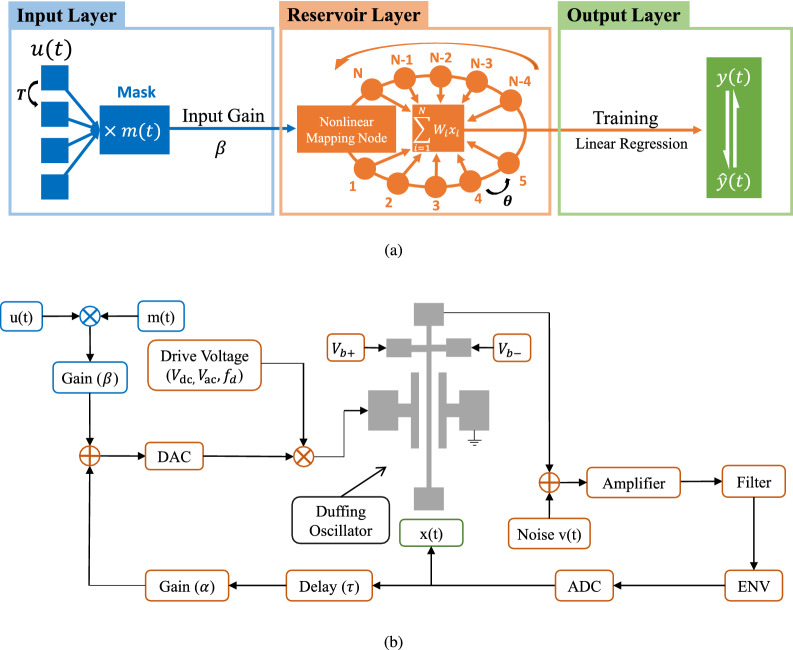
Schematic diagrams and signal chain of time-delay feedback RC structure. (**a**) Schematic diagrams. The blue part stands the input layer and orange part shows the Reservoir layer, while green part performs the output layer. (**b**) Signal chain for the simulation system. Before being supplied to the Duffing oscillator, the input signal goes through a digital to analog converter (DAC) and modulated by a sinusoidal drive signal. After processed by Duffing oscillator, the independent white Gaussian noise terms *v*(*t*) is injected, and the signal-to-noise ratio of the output is set to 20 dB. Response measurement involves bandpass filtering, the Envelope (ENV), and analog to digital converter (ADC). Before reinjecting back to DAC, the virtual node $x_i (n)$ processed by a delay $\tau$ and a feedback gain $\alpha$, in the Reservoir layer.

The time-delay feedback RC structure, as illustrated in Fig. 1a, is chiefly divided into three interrelated parts: the input layer, the Reservoir layer, and the output layer. It is theoretically proved that the three-part neural network with hidden states can remarkably approximate arbitrary continuous function infinitely^23,24^. The input layer injects the discrete or continuous input signal to the Reservoir layer through a fixed random connection, which is determined by a mask signal. Mask signal plays an important role in breaking the symmetry of the data and keeping the nonlinear nodes in the transient state to obtain various transient responses to the input data. Our mask signal consists of randomly chosen numbers in the range of [0,1] with zero mean and unit variance. Given the input signal *u*(*t*), $$nT\ll t<(n+1)T$$, the mask signal *m*(*t*),$$ m(t+T)=m(t)$$, and the input gain $$\beta $$, the signal injected in the Reservoir layer can be described as, $$I(t)=\beta *m(t)*u(t)$$. Specifically, the period *T* is composed of *N* segments which are called virtual node $$x_i (n)={\mathbb {R}} (i=1,\ldots ,N)$$, and the duration $$\theta $$, $$\theta =T/N$$, is the minimum time scale of RC system between two virtual nodes.

Inspired by Guillaume Dion’s pioneering research^21^, a single nonlinear node with a delayed feedback loop constitutes the Reservoir layer, as illustrated in Fig. 1b, which is the most crucial part of our system. The role of it is to transform the one-dimensional input data into spatiotemporal patterns in a high-dimensional space such that the features of the inputs can be efficiently read out by a simple learning algorithm. The current input and the delay virtual state of the Reservoir are superimposed to drive the nonlinear node into the next state. The input information is multi-dimensionally expanded based on the amplitude modulation with driving voltage as a carrier signal. The delay time $\tau$ and the delay feedback gain $\alpha$ determine the impact of the previous input signal on the current input signal. The independent white Gaussian noise term *v*(*t*) with zero mean and unity variance is included for the output of the Duffing oscillator. Virtual state is generated by bandpass filtering with central frequency $f_n$ which equals to driving frequency $f_d$ and sampling its envelope at a rate $\theta ^{-1}$ with a resolution of 12 bits. As have been proved in the previous study^25^, when $\tau =(N+k)T/N$, $1\ll k<N$, the system is in an unsynchronized state where the virtual node is connected with several dependent nodes in a fraction of period. Thus, we set $k=1$ to enrich the dynamic characteristics of the system. The output layer performs the training and testing part of the Reservoir system. The responses of the nonlinear mapping unit to the injected signal are linearly combined, $y(n)=\sum ^{N-1}_{i=0}W_{out}x_i(n)$, where $W_{out}$ is a set of readout weights, which are determined in the training process and remain constant for each virtual node during the entire processing task. The readout weights are trained by the ridge regression algorithm whose regularization parameter is to prevent overfitting.

### The sequence of parameters optimization

**Figure 2:**
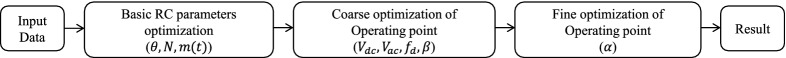
Flow chart of parameters optimization.

The simple structure of the time-delay feedback RC makes it amenable to hardware implementation without convoluted network of nonlinear physics nodes. In exchange, the system performance is sensitive to the architecture parameters that are highly dependent on the task at hand. More specifically, small deviations from the optimal parameters can seriously degrade the performance, and moreover, the architecture parameters are interrelated^26^. This problem makes the sequence of parameters optimization a very crucial step in the RC design. To tackle this difficulty, we divide these parameters into three parts in our optimization method, as illustrated in Fig. 2. The first part is the optimization of basic RC parameters that contains the duration of virtual nodes $\theta$, the number of virtual nodes *N*, and the mask signal *m*(*t*). The duration of virtual nodes $\theta$ is directly related to the decay time of the oscillator $T_d$ with the relation $T_d=a\theta$. The factor *a* indicates the interaction of current input to the previous virtual node state. When $a\ll 1$, the coupling strength between virtual nodes is too weak to interact with each other. When $a\gg 1$, each node does not have enough time to respond, which leads the system to an average state. Both cases result in performance degradation^7^. In many previous studies^11,12^, $T_d=5\theta$ was chosen to reach a good performance in different data sets. The number of virtual nodes *N* stands the dynamics of RC system. As *N* increases, the variety of dynamical units increases, which can be used to model a wider range of dynamic dependences^27^. In RC, a temporal mask is applied to each input data in order to introduce complex transient response under consistency conditions^28^. The mask signal we choose consists of randomly chosen numbers in the range of [0,1] with zero mean and unit variance.

After determining the basic parameters, we need to optimize the operating point of the system, which directly affects the system performance. Since the driving frequency and the driving amplitude (along with the feedback gain) essentially determine the nonlinearity in our system, whereas the driving frequency can be determined by the BPFM method we presented, finding the optimal driving frequency of the system is a top priority for optimizing the operating point in our research. So, the second part is the coarse optimization of the operating point with the BPFM method through, which consists of the driving voltage $V_{dc}$ and $V_{ac}$, the driving frequency $f_d$, and the input gain $\beta$. The details of the optimization are shown in the next section. Finally, the third part is the fine optimization of the operating point, optimizing the delay feedback gain $\alpha$ in a certain narrow range with the traditional parameter scanning method.

### The BPFM method

Hardware implementation of the Reservoir can be achieved using a variety of physical nonlinear nodes that can maps the injected data into a higher dimensional state in the real world, because a mechanism for adaptive changes for training is not necessary^29^. In this simulation, it is implemented using a single clamped–clamped silicon beam which is electrostatically driven at large oscillation amplitudes, as illustrated in Fig. 1b. For subsequent sillicon on glass (SOG) processing in our future work, the thickness of the silicon beam is set to be $40\,\upmu$m, while its in-plane length and width are chosen to be $500\,\upmu$m and $7\,\upmu$m, respectively. The natural frequency of this structure is about 350 kHz, which can ensure the high calculation speed of system. To implement the ‘fading memory’ property, which means the beam will forget the previous input rapidly, quality factor (*Q*) is set in a low range of about 500. And the gap for electrostatic driven is $2\,\upmu$m on the purpose of high efficiency. This system detects the mechanical displacement of the beam with piezoelectric materials. The beam’s mechanical displacement has a linear relationship with the output voltage, while the linear coefficient $k_{ec}$ is related to the size of the piezoresistive gage pairs (between the beam and bias electrodes):

1 $$\begin{aligned} V_{out}=k_{ec} x \end{aligned}$$

where *x* is the mechanical displacement of silicon beam. In our simulation, when the size of piezoresistive gage pairs is $10 \mu m \times 5\,\upmu {\text {m}} \times 40\,\upmu$m, linear convention coefficient $k_{ec}\approx 70$ V/m.

The dynamic part of the system is described by the following Duffing equation with forced harmonic vibration:

2 $$\begin{aligned} m\ddot{x}+c{\dot{x}}+k_1x+k_3x^3=Fcos(\omega t) \end{aligned}$$

where *m* is lumped effective mass, *c* is damping coefficient of system, $k_1$ is linear spring constant, $k_3$ is nonlinear spring constant which controls dynamic behavior of the beam, $\omega$ is driving frequency, *F* is harmonic force using the electrostatic drive. This higher-order ordinary differential equation can be solved by Multiple scale method^30^. Introducing small parameter $\varepsilon \ll 1$, Eq. (2) can be normalized as:

3 $$\begin{aligned} \ddot{y}+y+\varepsilon (2{\bar{\xi }}{\dot{y}}+{\bar{\gamma }}y^3)=\varepsilon \bar{F'}cos(\Omega t) \end{aligned}$$

where *y* is normalized displacement, $\Omega =1+\varepsilon \sigma$ is normalized driving frequency, $\sigma$ is called the detuning parameter which is a measure of how close the excitation frequency is to the natural frequency, ${\bar{F}}=\varepsilon \bar{F'}$ is the normalized harmonic force which is proportional to the driving voltage $v_b$, $\xi =\varepsilon {\bar{\xi }}$ is normalized damping coefficient, $\gamma =\varepsilon {\bar{\gamma }}$ is normalized nonlinear spring constant. Using the Multiple scale method, the frequency-response equation can be given as:

4 $$\begin{aligned} {\bar{F'}}^2=4a^2({{\bar{\xi }}}^2+(\sigma -\frac{3}{8}{\bar{\gamma }} a^2)^2) \end{aligned}$$

where *a* is the amplitude of beam. Condition for the existence of a fixpoint of the Duffing equation is the eigenvalue of the Jacobian matrix equals to zero. The condition equation is given as follows:

5 $$\begin{aligned} \Delta ={{\bar{\xi }}}^2+(\sigma -\frac{9}{8}{\bar{\gamma }}a^2)(\sigma -\frac{3}{8}{\bar{\gamma }}a^2)=0 \end{aligned}$$

From Eqs. (4) and (5), it follows that:

6 $$\begin{aligned} {\bar{F'}}^2=3{\bar{\gamma }}a^4(\sigma -\frac{3}{8}{\bar{\gamma }}a^2) \end{aligned}$$

According to Eq. (6), the bifurcation point can be obtained at the point where the partial derivative of $\bar{F'}$ with respect to variable *a* is zero, $\frac{\partial \bar{F'}^2}{\partial a}=\frac{\partial \bar{F'}}{\partial a}=0$. Using this condition, we can obtain the equation below:

7 $$\begin{aligned} {\bar{F'}}^2=\frac{256\sigma ^3}{81{\bar{\gamma }}} \end{aligned}$$

For the case when the nonlinearity and the detuning of the system are small, and consider that ${\bar{\gamma }}$ and $\sigma$ are both dimensionless quantities, Eq. (7) can be written as:

8 $$\begin{aligned} {\bar{F'}}=\pm \frac{16(\Omega -1)}{9\varepsilon } \end{aligned}$$

It means that the driving voltage of the bifurcation point has a linear relationship with the driving frequency:

9 $$\begin{aligned} v_b=k_bf+b \end{aligned}$$

where $k_b$ is the linear bifurcation shift coefficient, and *b* is the constant term.

**Figure 3:**
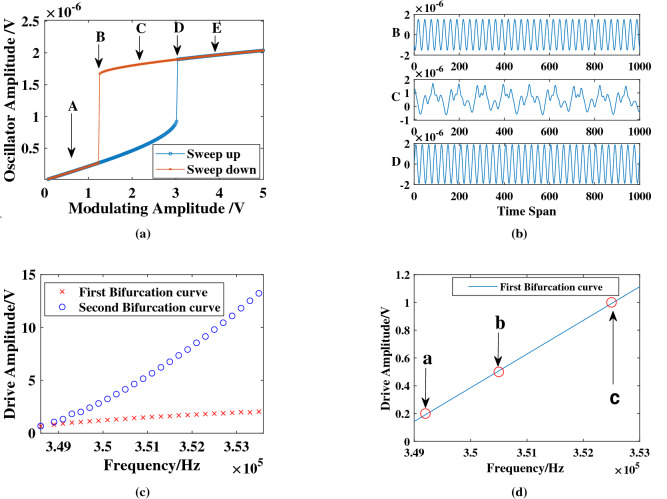
Bifurcation analysis of Duffing oscillator. (**a**) The response of the oscillator to an amplitude modulated signal for a fixed drive frequency of 350 kHz. Blue line indicates the sweep-up curve while orange line indicates the sweep-down curve. The hysteresis phenomenon depended on the amplitude sweep represents the strong nonlinear response of the resonator. In our research, we drive the resonator to the edge of the first bifurcation point. (**b**) The output signal of the Duffing oscillator with a fixed input signal in three different modulating amplitude. (**c**) Bifurcation point shift with the driving frequency. The red cross represents the first bifurcation curve and the blue circle represents the second bifurcation curve. (**d**) The linear relationship between the driving frequency and driving amplitude in the first bifurcation. Point a, Point b and Point c are the operating points in three different tasks.

**Figure 4:**
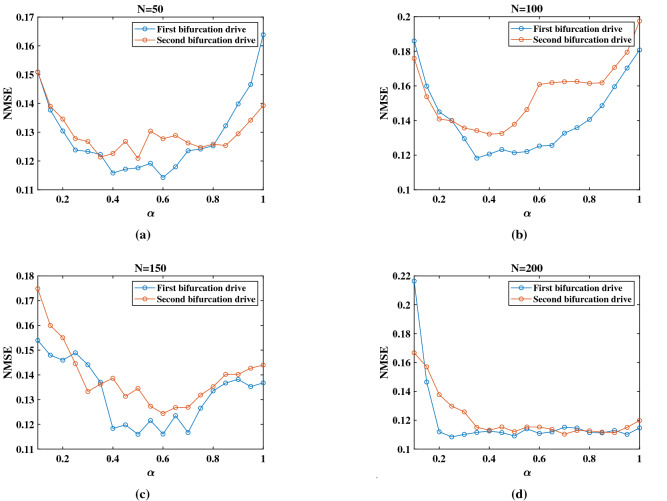
NMSE as a function of delay feedback gain for different virtual nodes N.

With a fixed driving voltage, using this linear relationship is an efficient way to find the bifurcation region of the oscillator. Since the RC system deals with the time-dependent signal and the input signal injected into the system is modulated by the amplitude, the response of the system to a driving amplitude sweep for a fixed driving frequency is critical to our research. Figure 3a shows the simulation results of the response of the oscillator to an amplitude modulated signal for a fixed driving frequency of 350 kHz. Five typical modulating amplitude points (A–E) are shown in the figure. Among these five points, Point A and Point E represent the points away from the hysteresis region, which exhibits such weak nonlinearity that is not applicable for this nonlinear system. Figure 3b shows the outputs of the Duffing oscillator that are driven at Point B, Point C, and Point D for a fixed modulating signal. The output of Point C which between two bifurcation points is so unstable and irregular that it cannot be used as a stable carrier to modulate the input signal. In contrast, Point B and Point D at the two bifurcation points are relatively stable. In order to choose the better point from Point B and Point D, several experiments are performed. Apparently, the results of the NARMA10 task that are shown in Fig. 4 (the details of this task are explained below) reveal that the NMSE of the first bifurcation (Point B) is better than that of the second bifurcation (Point D) for different virtual node *N* and different delay feedback gain $\alpha$. And the results of the Parity benchmark task are shown in the Supplemental Information 1. These results prove that the first bifurcation (Point B) is the optimal operating point of our RC system. According to the BPFM method, we can find the corresponding driving frequency of the Point B for different driving amplitude determined by the input. Figure 3c shows the quasi-linear relationship between the driving frequency and the driving voltage of the bifurcation point for the case of weak nonlinearity and small detuning of the system. According to the Multiple Scale Method, the smaller the detuning of the system is (that is, the driving frequency is closer to the natural frequency), the more obvious the linear relationship is. To ensure this quasi-linear relationship, the range of frequency scanning is limited from 345 to 355 kHz.

## Results

The performance of the Reservoir computing system is sensitive to its optimizable architecture parameters, such as driving frequency, driving amplitude, input gain, and feedback gain. The parameters are not some specific values applicable to each data set, but some reasonable range needs to be adjusted. The selection of the parameters adjustment sequence and the determination of the parameters range directly affects the performance of the Reservoir system.

**Figure 5:**
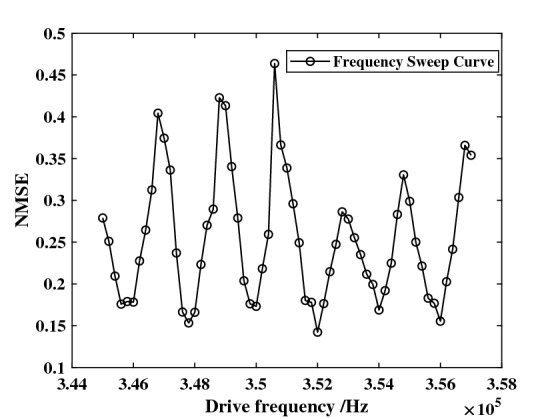
Driving frequency sweep of NARMA10 test. The smallest NMSE appears while driving frequency is 352 kHz.

Essentially, the system should be set to the optimal operating point to achieve good performance for a given data set. The coarse operating point is decided by input gain, driving amplitude, and driving frequency. But up to now, no research has given an efficient way to find the operating point. The parametric scanning method of finding the optimal operating point is time-consuming and less precise. To be specific, after determining driving amplitude, a frequency scanning result of the NARMA10 prediction test (see below) is shown in Fig. 5. The Normalized Mean Square Error (NMSE) result is quite unstable in this small range. So, the range of frequency scanning must be extended to get the optimal driving frequency. Moreover, this kind of scanning requires so much more computing capacity that it is difficult to implement in a hardware system.

This article proposes a novel method to optimize the operating point based on the bifurcation point shift caused by the change of driving frequency, which is called Bifurcation Point Frequency Modulation (BPFM). The driving amplitude which determines the nonlinear strength of the system is the first parameter that needs to be determined. When driving amplitude is determined, the linear coefficient $k_b$ and *b* are fixed at the same time. Through Eq. (9), utilize the maximum value of the input data from different data sets as the driving amplitude to determine the value of the driving frequency of each different task. The maximum data value located at the edge of the bifurcation region ensures that most of the input data is located around the bifurcation region.

There are two crucial aspects to measure the performance of RC system. The prediction performance is numerically investigated via Parity benchmark prediction task and NARMA10 task, while the classification performance is discussed via signal classification task and TI-46 isolated word task.

### Signal classification task

To evaluate the classification performance of the RC system, the signal classification task is taken as an example. Waveform classification is a relatively simple task because its linearly separable. The input signal of this task is taken to be a random combination of sine and square waves which is discretized into 10 points per period, and the target function *y*(*n*) is 1 for a sine wave and 0 for a square wave. In this simulation, we choose $T_d=5\theta$, and $\theta =0.1ms$. Since this data set is simple, not many virtual nodes, here we choose $N=50$, can achieve a high enough accuracy rate. The driving amplitude $V_{dc}=20V$, $V_{ac}=2V$ can determine the linear coefficient $k_b=2.424e-4$ and the constant term $b=-84.4545$. The input signal of this task is limit to the range of [0,0.5] when the input gain $\beta =1$, thus, the optimal driving frequency should be around 350500Hz according to equation (9). The operating point of this task corresponds to the Point b in Fig. 3d. And then, determine $\alpha =0.01$ through the scanning parameters method.

**Figure 6:**
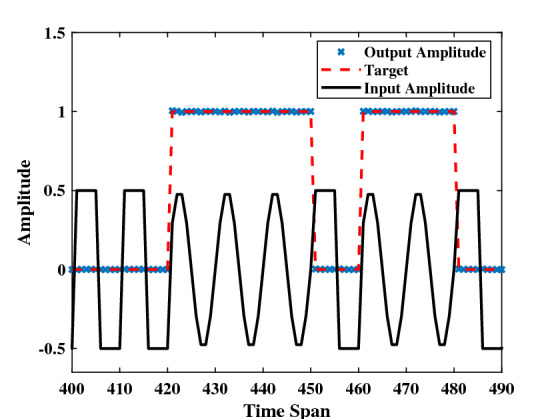
Performance illustration of the signal classification task. This task aims to differentiate between square and sine waves. The target function (dashed line) equals to 1 when the input signal is a sine function and equals to 0 when the input signal is a square function. After evaluating over 1000 inputs, NMSE = 1.2e−4.

As illustrated in Fig. 6, the black line represents the input *u*(*t*) after sampling and holding circuit. The output of the Reservoir system after training is indicated by blue crosses, against the desired output represented by a red line. The performance on this task is essentially perfect: the $\text {NMSE}=\frac{1}{L}\sum _{n=1}^{L}(y(n)-{\hat{y}}(y))^2/var(y)$ is up to 1.2e−4, which is significantly better than 1.5e−3 reported in^25^ and 5.5e−4 reported in^31^.

### Parity benchmark prediction task

**Figure 7:**
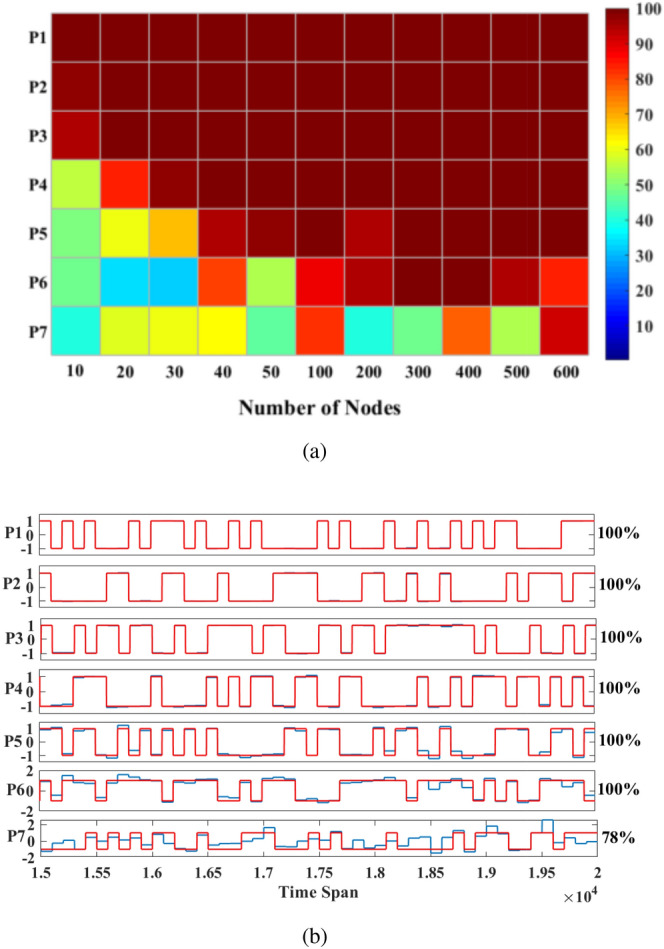
Results of the Parity Benchmark test. (**a**) The success rate as a function of the number of virtual nodes. The color bar on the right indicates the percentage of success rate. (**b**) Performance of the system for the Parity benchmark test. From top to bottom, waveforms show the prediction result of the system *y*(*t*) (blue) overlaid on the target $y'(t)$ (red) in the time span of [1.5e4, 2e4] after the training phase for $P_n$ with $n=1,2,3,4,5,6,7$. Success rates for the individual tasks, evaluated for 2000 timesteps of the input function, are indicated on the right.

The Parity benchmark prediction task proves the prediction ability of Reservoir system. A random binary sequence *u*(*t*), with values drawn from [− 1, 1], is fed into the Reservoir system. The $n^{th}$-order parity function can be written as:

10 $$\begin{aligned} P_n=\prod _{i=1}^{n-1}u(t-i\tau ) \end{aligned}$$

where $P_n$ is the training target of $n^{th}$-order benchmark. When $n=1$, this benchmark represents a linear data set which is uncomplicated to predict. When $n \ge 1$, the parity function is not linear separable^27^ and depends on the input at times $t-i\tau$. Compared with the similar research^22^ and^21^, we achieved better results using only 150 training data and 50 testing data. In this task, we choose the parameters, $\theta =0.1ms$, and $V_{dc}=20V$,$V_{ac}=2V$ for the better success rate. Figure 7a shown that, when the duration of virtual nodes $\theta$ is given as 0.1 ms, the success rate is a function of the number of virtual nodes *N*. The success rate of $n\le 6$ gradually stable at $N=400$, while larger *N* will decrease the final result. Since decay time of the oscillator $T_d=5\theta$, it indicates that the system does not have enough memory length. So, the success rate of $P_7$ which needs more memory capacity is quite unstable. Under the condition that the input signal range of this task is [− 1,1] when the input gain $\beta =1$, the optimal driving frequency should be around 352,500 Hz. The operating point of this task corresponds to Point c in Fig. 3d. Using the scanning parameters method, we obtain the optimal delay feedback gain, $\alpha =5$. Figure 7b shows the system’s prediction overlaid on the target Eq. (10) for n = 1, 2, 3, 4, 5, 6, 7. ( The difficulty of system predication increases as n increases.) Red lines show the output target while blue lines stand the Reservoir result. Success rates for individual tasks are indicated on the right. The success rate is 100$\%$ when $n\le 6$. For the case of $n=7$, the Reservoir system still gives a near bit-perfect reproduction of the target, but the output signal becomes noisy because of the increased difficulty and the memory requirement of the task. Besides, we performed a frequency scanning experiment in the Supplementary Information 1 to prove the feasibility of our BPFM method. And the results show the accuracy of this method for searching the optimal driving frequency for a given task.

### TI-46 digit word classification task

In order to verify the recognition performance of the Reservoir computer, we optimized parameters in a more complex task. We perform a benchmark task called spoken word recognition that is common in the Reservoir computing community. In this task, the input dataset consists of a subset of the National Institute of Standards and Technology Texas Instrument-46 Corpus (NIST TI-46 Corpus) with ten spoken digits (0–9) pronounced ten times by five different female speakers separately^32^. The chosen part of the database contains 500 (5 speakers $\times$ 10 digits $\times$ 10 utterances) audio files, which are sampled at a rate of 12.5 kHz with variable time lengths. To extract the acoustic features contained in the frequency information effectively, each input that represents a spoken digit is preprocessed by a standard Lyon cochlear ear model^33^ before it is fed into the RC. For the purpose of the classification, ten linear classifiers are trained for the ten digits in the vocabulary respectively. Each classifier should be set to 1 if the utterance of the digit corresponds to the desired digit, and 0 otherwise. The results of the classifiers are averaged in time and a winner-takes-all approach that the highest averaged classifier corresponds to the correct category is applied to select the actual digit during the training phase. Then, a performance metric called Word Error Rate (WER) is used to evaluate the recognition task, which is the fraction of digits incorrectly classified. As the subset corpus has only 500 sequences, we divide them into ten parts and estimate them subsequently with the ten-fold cross-validation procedure to minimize the fluctuations that occur in the results due to random choices between the training and testing sets. Ten parts are chosen randomly, one of which is used for testing and nine for training.

In this task, the number of virtual nodes *N* is set to be 100 for the sake of the calculation efficiency, and the duration of virtual nodes $\theta$ is set to be 0.1*ms*. Same as the previous two experiments, we choose the driving amplitude $V_{dc}=20V$, $V_{ac}=2V$. The magnitude of the input audio signal after processed by the Lyon cochlear ear model and input mask is quite small, about [0,1e−3]. Therefore, setting input gain $\beta =200$ could convert the input range into [0,0.2]. According to Eq. (9), the optimal driving frequency is around 349,200 Hz. The operating point of this task corresponds to Point a, as shown in Fig. 3d. When setting feedback gain $\alpha =0.4$, the best classification result WER=0.2$\%$ can be obtained which corresponds to the state-of-the-art. Compared with previous results, the traditional RC^34^ gets WER = 4.3$\%$ with more than 1200 nodes and using the more complicated electronic system. Nanoscale spintronic oscillators with 400 virtual nodes achieve an error rate of $0.4\%$, and $0.4\%$ at $N = 200$ in the optoelectronic RC with single feedback loop^25^. Besides, we performed a training part experiment of this data set in the Supplementary Information 1.

**Figure 8:**
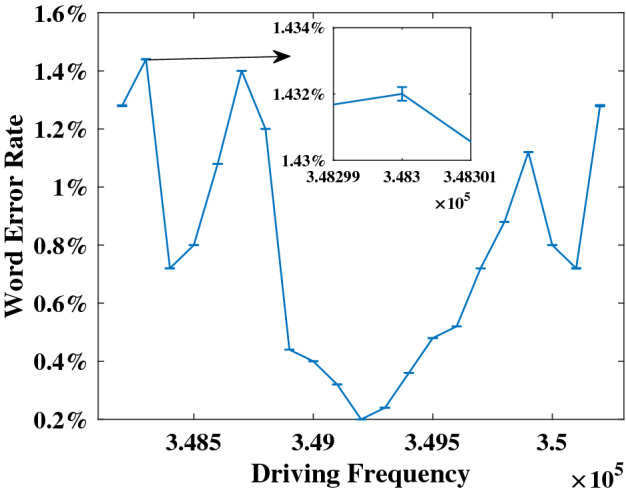
Frequency scanning results of the Ti-46 word classification task. Error bars show the standard deviation for 5 trials.

Figure 8 shows the WER as a function of driving frequency. The smallest WER appears at the driving frequency $f_d=349{,}200\,\hbox {Hz}$ that is consistent with our above result obtained with the BPFM method. As the driving frequency deviates from the optimal value, the WER increases and fluctuates obviously, which proves the significance of the optimal operating point for the RC system.

### NARMA10 prediction task

The NARMA10 task is one of the most popular prediction tasks in the RC community. In this task, our Reservoir system will be trained to predict the behave of the system, like a nonlinear auto regressive moving average equation of order 10 driven by white noise. The NARMA10 task is given by the following recursive formula:

11 $$\begin{aligned} y(n+1)=0.3y(n)+0.05y(n)\left( \sum _{i=0}^{9}y(n-i)\right) +1.5u(n-9)u(n)+0.1 \end{aligned}$$

where *u*(*n*) is the random input drawn from a uniform distribution over the interval [0, 0.5] which stands white noise and *y*(*n*) is the output target of the system. In this task, the RC is trained with a sequence of 1000 time steps and tested over a subsequent sequence of 1000 time steps. The performance vector used to evaluate the NARMA10 is the NMSE.

NARMA10 is a more difficult task compared to those three above, which needs ten memory steps as well as a more complicated dynamic property. In this task, the number of virtual nodes *N* is set to be 50 for compromising between calculation time and NMSE. The details of the number of virtual node test are shown in Supplementary Information 1. The duration of virtual nodes $\theta$ should be set to 0.01*ms* to satisfy the relationship of $T_d=50\theta$, which enhanced the connection of current input to previous virtual node state. To enrich the mapping dimension of the reservoir system, the nonlinear spring constant $k_3$ should be increased appropriately by setting the driving voltages, $V_{dc}=80V$, and $V_{ac}=2V$. Then, we can reconstruct the coefficients, $k_b=0.000256$ and $b=-88.112$, using the simulation method of equation (9). The input signal of this task is limited to the range [0,2] when the input gain $\beta =3.8$, which indicates that the optimal driving frequency should be around 352,000 Hz. The best result for this task is NMSE=0.114 when we choose the delay feedback gain $\alpha =0.6$. Compared with previous results, the NMSE = 0.168 was obtained in^25^, and the NMSE = 0.152 in^35^ with the same number of virtual nodes.

**Figure 9:**
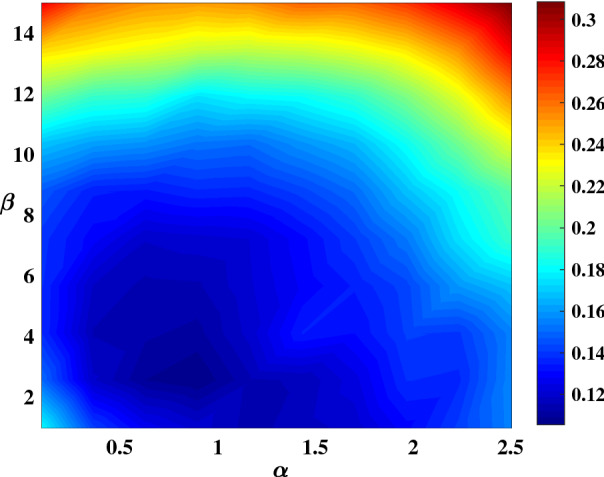
NMSE for the NARMA10 in the input gain-delay feedback gain plane. The fixed parameters are $\theta =0.01ms$.

Figure 9 shows the NMSE as a function of delay feedback gain and input gain at a fixed driving frequency. A large region with NMSE $< 0.12$ has been obtained. In the dark blue area, the Reservoir system can get reasonable results. Thus, we achieved comparable performance to conventional RC using this parameters optimization method. The frequency scanning experiment of this prediction task is also shown in the Supplement Information 1.

## Conclusion

We propose a novel method called Bifurcation Point Frequency Modulation (BPFM) to find the optimal driving frequency and put forward a comprehensive optimization process for the time-delayed Reservoir computing with a nonlinear Duffing mechanical oscillator. BPFM describes the relationship between the driving voltage of the bifurcation point and the driving frequency near the resonant frequency. According to this method, we find the optimal driving frequency of the RC described in this article for different tasks. Then we optimize the system according to the optimization process we put forward. And we numerically analyze its performance via some typical tasks such as the signal classification, the parity benchmark prediction, the TI-46 digit word classification, and the NARMA10 prediction. The simulated results show that the NMSE is 1.2e−4 for the signal classification, the success rate is 100$\%$ at $P_1\sim P_6$ and 78$\%$ at $P_7$ for the Parity benchmark prediction, the WER is 0.2$\%$ for the TI-46 digit word classification, the NMSE is 0.114 for NARMA10 predication. The high performance proves that the method based on BPFM can be used to find the bifurcation of the Duffing oscillator, which greatly improves the efficiency of optimizing the operating point for the RC. Moreover, we expect that the method based on BPFM can facilitate our future work on hardware implementation of the time-delayed RC with other mechanical oscillators.

## Supplementary information

Supplementary information.
