# Supplementary material for: Parameters optimization method for the time-delayed reservoir computing with a nonlinear duffing mechanical oscillator
Source: Sci Rep. 2021 Jan 13;11:997. doi: 10.1038/s41598-020-80339-5 (PMC7806606; doi:10.1038/s41598-020-80339-5)
Supplement: Supplementary file 1 — Supplementary information. [file 41598_2020_80339_MOESM1_ESM.pdf]

Supplementary Information: Parameters Optimization Method for the Time-delayed Reservoir Computing with a Nonlinear Duffing Mechanical Oscillator

T. Y. Zheng[1,2], W. H. Yang[1], J. Sun[1,2], X. Y. Xiong[1], Z. T. Li[1], and X. D. Zou[1,2,*]

[1]The State Key Laboratory of Transducer Technology, Aerospace Information Research Institute, Chinese Academy of Sciences, Beijing, CHINA

[2]School of Electronic, Electrical and Communication Engineering, University of Chinese Academy of Sciences, Beijing, CHINA

1. Bifurcation analysis of two Point

In order to choose the optimal point between the two bifurcation Point B and Point D in Figure 3 (b). We compare the impact on the performance of the RC we proposed for the two cases that the oscillator was driven to the first bifurcation B and second bifurcation D respectively. Two different data sets, the Parity benchmark task and the NARMA10 task, are used for the analysis. The results of the NARMA10 task have been shown in the main text, and the results of the Parity benchmark task are shown here.

In the Parity benchmark task, for the low order cases n equals to 1~5, the success rates are too high to distinguish from each other, so we utilize the cases $n = 6$ and $n = 7$ to analyze the impact on the performance when the oscillator was driven to the Point B (first bifurcation) and Point D (second bifurcation). Supplementary Figure S1 shows that the success rate varies with different delay feedback gain α for different virtual nodes N. For the convenience of comparison, the parameters are set as $T_d = 5\theta$, $V_{dc} = 20V$, $V_{ac} = 2V, \theta = 0.1ms, f = 352500$Hz. The results show that the success rate of two points increases with an increasing N. Moreover, for all the four cases, the success rate of Point B is better than that of Point D for the same virtual nodes N.

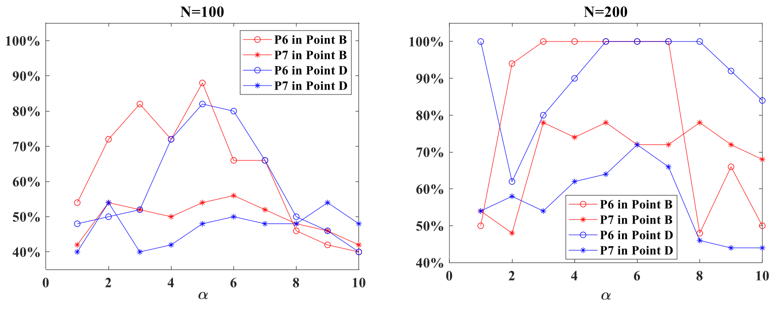

(a) (b)

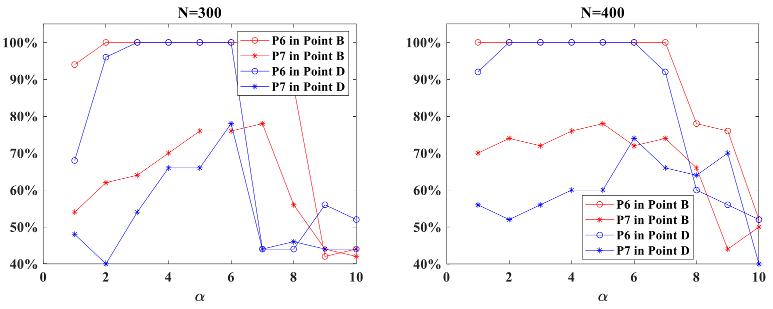

(c) (d)

Supplementary Figure S1. The success rate as a function of delay feedback gain for the cases $n = 6$ and $n = 7$.

2. Frequency scanning experiments of two data sets

As described in section III.B, we have obtained the optimal driving frequency that is around 352500Hz with our BPFM method in the Parity benchmark prediction task. In order to verify the feasibility of this method, we perform the frequency scanning simulation to search the optimal driving frequency under the same condition as the comparison. Supplementary Figure S2 shows the result of the frequency scanning when we set the parameters as $N = 400$, $T_d = 5\theta$, $V_{dc} = 20V$, $V_{ac} = 2V$, $\theta = 0.1ms, \beta = 1, \alpha = 5$ in the 6-th and 7-th order of parity benchmark. In both cases, the highest success rate appears at an optimal driven frequency $f_d = 352500Hz$ that is consistent with the result obtained with the BPFM method. More specifically, as the deviations from this optimal frequency increase, the success rate seriously degrades. For the case of $n = 7$, the success rate degrades more obviously than the case of $n = 6$ because of the increased difficulty and the memory requirement of the task.

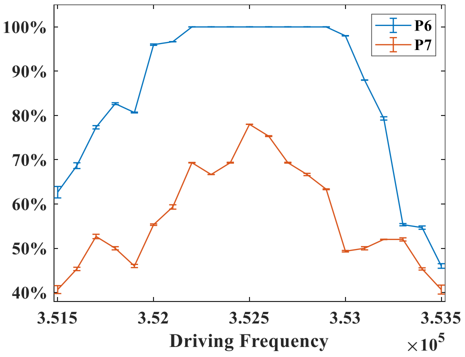

Supplementary Figure S2. Frequency scanning results of the Parity benchmark task. Error bars show the standard deviation.

In the NARMA10 predication task, the optimal driving frequency lies at $f_d = 352000$Hz according to the frequency scanning, which is in line with the result obtained using the BPFM method under the same condition $N = 50$, $T_d = 50\theta$, $V_{dc} = 80V$, $V_{ac} = 2V, \theta = 0.01ms, \beta =$

3.8, $\alpha = 0.6$. The NMSE (Normalized Mean Square Error) as a function of driving frequency is shown in Supplementary Figure S3. As the drive frequency deviates from the optimal value 352000Hz, the NMSE increases obviously.

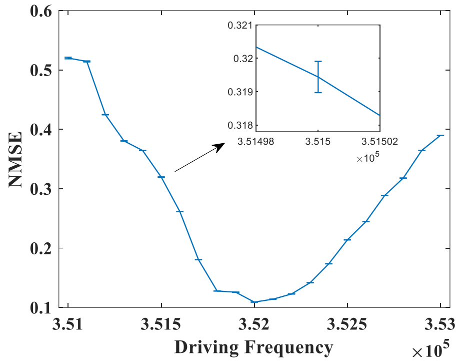

Supplementary Figure S3. Frequency sweep results of the NARMA10 prediction task. Error bars show the standard deviation.

3. Number of virtual nodes optimization with NARMA10 task

The NMSE as a function of the number of virtual nodes is shown in Supplementary Figure S4 for the NARMA10 task. The other parameters are set as $f_d = 352000$Hz, $T_d = 50\theta$, $V_{dc} = 80V$, $V_{ac} = 2V$, $\theta = 0.01ms$, $\beta = 3.8$, $\alpha = 0.6$. As the number of virtual nodes increases, the NMSE decreases significantly to an asymptotic value for $N \geq 50$. However, the simulation time of the RC system increases remarkably with the increasing N. As a compromise between the performance and the efficiency, we choose N=50 in this task.

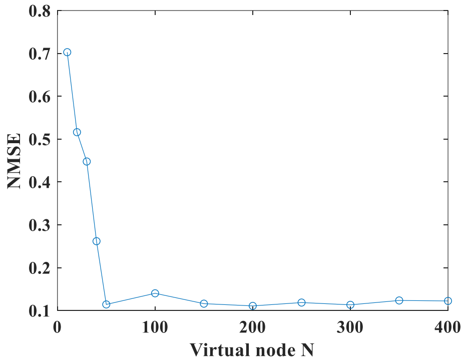

Supplementary Figure S4. Virtual node sweep results of the NARMA10 prediction task.

4. Number of training data test of TI-46 task

The details of the training data test in the TI-46 task are introduced here. Supplementary Figure S5 shows the WER of the Reservoir system as a function of the number of training data parts. In

this task, we have a total of ten parts of data, some of which are used as the training parts, and the others are used as the testing parts. The simulation results are consistent with common sense that the WER will decrease as the number of training parts increasing. Obviously, using nine parts for training will get the best result.

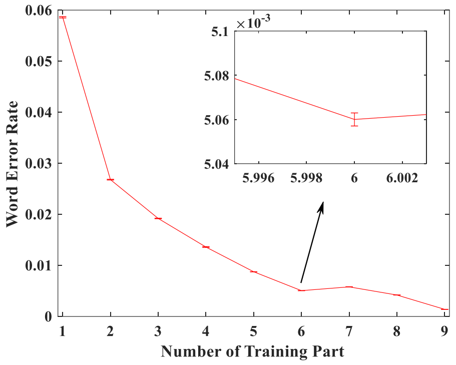

Supplementary Figure S5. Word error rate as a function of the number of training parts. Error bars are the standard deviation for 5 trials.
